# Association Between Changes in Shoulder Strength and Self‐Reported Shoulder Symptoms in Patients With Hypermobility Following 16‐Weeks of High‐Load or Low‐Load Exercise: A Secondary Analysis of an RCT

**DOI:** 10.1002/pri.70197

**Published:** 2026-03-18

**Authors:** Thomas Christensen, Carsten Bogh Juhl, Birgit Juul‐Kristensen, Søren T. Skou, Jens Søndergaard, Karen Søgaard, Behnam Liaghat

**Affiliations:** ^1^ Center for Muscle and Joint Health Department of Sports Science and Clinical Biomechanics University of Southern Denmark Odense Denmark; ^2^ Department of Physiotherapy and Occupational Therapy Copenhagen University Hospital Herlev and Gentofte Denmark; ^3^ The Research and Implementation Unit PROgrez Department of Physiotherapy and Occupational Therapy Central and West Zealand Hospital Slagelse Denmark; ^4^ Department of Research Central and West Zealand Hospital Slagelse Denmark; ^5^ Research Unit for General Practice Department of Public Health University of Southern Denmark Odense Denmark; ^6^ Department of Clinical Research University of Southern Denmark Odense Denmark

**Keywords:** joint instability, randomised controlled trial, resistance training, shoulder

## Abstract

**Background and Purpose:**

Hypermobility Spectrum Disorder (HSD) is a common musculoskeletal condition that impairs function and quality of life. While exercise therapy has shown to improve outcomes, treatment standardisation is lacking and the relationship between strength and symptoms remains unclear. Therefore, our aim was to investigate the association between changes in shoulder strength and self‐reported shoulder function, pain, and perceived effect in patients with HSD and shoulder symptoms.

**Methods:**

This is a secondary analysis of a randomised controlled trial. Data from 76 participants (58 women; median age 35.5) were included. Primary outcomes were percentage change in relative strength (Nm/kg) measured in external rotation, internal rotation, and scaption, the Western Ontario Shoulder Instability Index (WOSI), the Numerical Pain Rating Scale (NPRS) and the Global Perceived Effect: Physical domain (GPEP). Secondary outcomes were achieving the Minimal Clinically Important Difference (MCID) in shoulder function and pain. Covariates were age, sex, Body Mass Index, hand dominance, previous shoulder dislocation, mechanical shoulder symptoms, and assigned intervention group.

**Results:**

Increasing strength in external rotation and scaption were linearly associated with an improvement in shoulder function (WOSI adjusted −3.5 (95% CI −5.9; −1.1), and WOSI adjusted −2.6 (95% CI −4.6; −0.6) respectively), while scaption strength was associated with a reduction in shoulder pain (NPRS −0.01 (95% CI −0.02; 0.00)). Improvement in external rotation and scaption were associated with higher odds of reporting an important improvement measured for every 10% increase in strength (adjusted OR 1.18 (95% CI 1.00; 1.38) and 1.30 (95% CI 1.07; 1.59), respectively).

**Discussion:**

Increased shoulder strength may be associated with improved outcomes in patients with HSD and persistent shoulder symptoms although our findings suggest limited clinical relevance. Further research is needed to understand the relationship between strength and symptom relief.

**Trial Registration:**

The study was registered in Clinicaltrials.gov (11 March 2019, NCT03869307).

## 1 Introduction

Generalised Joint Hypermobility (GJH) is a hereditary condition characterised by an excessive joint range of motion. The prevalence in Denmark is estimated to be 30% (Juul‐Kristensen et al. 2017); however, the prevalence varies from 2% to 57% depending upon population demographics, testing methods, and diagnostic criteria (Remvig et al. 2007). Hypermobility Spectrum Disorder (HSD) is a symptomatic subgroup of GJH, in which patients experience one or more secondary musculoskeletal symptoms (Castori et al. 2017). At least four out of five patients with HSD report impaired shoulder function, shoulder pain and reduced health‐related quality of life compared to the general population (Johannessen et al. 2016; Kalisch et al. 2020; Scheper et al. 2016). Furthermore, patients with HSD have higher rates of injury and may experience co‐morbidities such as anxiety (Liaghat et al. 2021; Sanches et al. 2012; Sienko‐Awierianow and Chudecka 2020), adding to the difficulty of treatment in a health care setting.

Exercise therapy is commonly recommended in treating patients with HSD and persistent shoulder symptoms; however, intervention studies often contain large heterogeneity, and the standardisation of exercise modality and intensity is lacking (Engelbert et al. 2017; Palmer et al. 2014; Warby et al. 2014). Thus, improving our understanding of which modifiable variables are associated with improved outcomes may provide valuable insight for further research and lay the groundwork for improving the clinical relevance of prescribing exercise.

A useful tool in the rehabilitation of shoulder conditions is resistance training for the purpose of strengthening the scapular, deltoid, and rotator cuff muscles (Eshoj et al. 2020; Ingwersen et al. 2017; Warby et al. 2018), yet uncertainty about what type of exercise to offer patients with HSD remains (Palmer et al. 2014; Warby et al. 2014). Considering that loading at higher intensities has been shown to effectively increase muscle and tendon strength (Couppe et al. 2008; Laudner et al. 2013), this training methodology may be of value to patients with HSD as they often display mechanical strength impairments of the shoulder (Alsiri et al. 2019; Coussens et al. 2022; Rombaut et al. 2012). Despite the potential benefit, the development of strength through higher load exercise has typically been avoided when treating patients with hypermobility, in part due to safety concerns (Liaghat, Skou, Jørgensen, et al. 2020; Liaghat, Skou, Sondergaard, et al. 2020).

A recent randomised controlled trial (RCT) by Liaghat et al. 2022b compared the use of high‐load and low‐load shoulder exercises in patients with HSD. While both interventions resulted in clinically meaningful improvements in shoulder function at the 16‐week follow‐up, an absolute between group difference of 8.3% favoured the high‐load group in an intention‐to‐treat analysis (Liaghat et al. 2022b). These results suggests that high‐load exercise for patients with HSD may be more efficacious in improving symptoms compared with traditional low‐load exercise, although the study did not examine the extent to which changes in strength were associated with improved outcomes. Although strength deficits are often present in patients across a variety of musculoskeletal conditions (Clausen et al. 2017; Sousa et al. 2019; Deasy et al. 2016), evidence on the association between strength and symptoms is conflicting (Clausen et al. 2018, 2017; Ingwersen et al. 2017; Oh et al. 2019), and few studies have examined strength improvement as a single modifiable variable, thus warranting further investigation.

Therefore, the aim of this study was to investigate the association between relative changes in shoulder strength and self‐reported shoulder function, pain, and perceived effect in patients with HSD and persistent shoulder symptoms.

## 2 Methods

### 2.1 Study Design

This study is a post hoc exploratory analysis using data from an RCT (Liaghat et al. 2022b), examining the effect of 16 weeks of shoulder exercise in either a high load (HEAVY) or low load (LIGHT) intervention group on patients with HSD and shoulder symptoms for more than 3 months. The RCT was approved by the Regional Committees on Health Research Ethics for (31 May 2017, S‐20170066) and registered in Clinicaltrials.gov (11 March 2019, NCT03869307). Data from participants who completed testing at both baseline and 16‐week follow‐up were included and analysed in this study, reported according to STROBE guidelines (Elm et al. 2008).

### 2.2 Setting

The RCT was conducted as a two‐group multicentre study. A project manager was responsible for the randomisation process and practical management of the project, and a total of 23 physiotherapists delivered the interventions. Outcome assessment was completed at two outpatient departments by four blinded trained physiotherapists not otherwise affiliated with the project.

### 2.3 Participants

A total of 100 participants were recruited from primary care within the Region of Southern Denmark between March 2019 and September 2020, representing a general patient population in Denmark, with the primary endpoint being post‐intervention at 16‐week follow‐up. Of these participants, 76 were included in this analysis having complete data at baseline and 16 weeks. All data were handled and saved anonymously to ensure privacy while adhering to the guidelines of the Danish Data Protection Agency.

### 2.4 Eligibility

Patients were eligible if aged 18–65 years with HSD, which included musculoskeletal manifestations (i.e., persistent shoulder pain and/or shoulder instability symptoms for at least 3 months without any cut‐off for symptom intensity) and the presence of GJH. Generalised HSD (G‐HSD) was defined by Beighton score cut off ≥ 5/9 for females up to the age of 50 years, and ≥ 4/9 for females > 50 years and males (Malfait et al. 2017). Additionally, Historical HSD (H‐HSD) was used for eligibility when the Beighton score was one point below the age and sex‐specific cut off, and where the 5‐part hypermobility questionnaire (5PQ) was positive (≥ 2/5 positive answers) (Hakim and Grahame 2003). Patients were excluded if experiencing co‐morbidities such as systemic inflammatory rheumatic diseases, connective tissue diseases (except hypermobile Ehlers‐Danlos Syndrome), neurological diseases or shoulder surgery within the last year (Liaghat, Skou, Sondergaard, et al. 2020). For this secondary analysis, patients were included if they had completed both pre‐ and post‐testing in strength and symptom measurements. We included only the shoulder with the most severe symptoms for each patient. To our knowledge, none of the included patients systematically used drugs that could affect shoulder joint strength or function, and none participated in other experimental studies.

### 2.5 Intervention

Both groups received education on scapular correction and general advice on joint protection adapted by the Danish Rheumatism Association.

#### 2.5.1 High‐Load Exercise (HEAVY)

Participants randomised to HEAVY received 16 weeks of progressive resistance training, twice a week supervised at a physiotherapy clinic (60 min for first session, 30 min for following sessions) and once a week (non‐supervised) at home/self‐selected location. The program included five exercises: side lying external rotation (ER) in neutral, prone horizontal abduction, prone ER at 90° of shoulder abduction, supine scapular protraction, and seated shoulder elevation in the scapular plane. A 10 repetition‐maximum (RM) was estimated using Brzycki's formula and loads were progressively increased over time with 50% of 10 RM at week 1, 70% of 10 RM at week 2 and 90% of 10 RM at week 3. The following 6 weeks (weeks 4–9) included 3 sets of 10 RM. From weeks 10–15, the training included 4 sets of 8 RM (Liaghat et al. 2022b). A tapering period was applied in week 16 to allow for the anabolic response before follow‐up testing.

#### 2.5.2 Low‐Load Exercise (LIGHT)

Participants randomised to LIGHT received 16 weeks of home‐based exercise, three times a week, performed at home/self‐selected location. Participants had an individual face‐to‐face introduction before initiating the program, as well as individual supervision at week 5 and week 11 as new exercises were introduced (30 min per session). The program included nine exercises with; phase 1 (isometric), posture correction; phase 2 (isometric), shoulder abduction, shoulder internal and external rotation with 90° flexion at the elbow joint and standing with the shoulder and arm against a wall; and phase 3 (dynamic with resistance band), shoulder abduction, shoulder internal and external rotation at 90° flexion at the elbow joint, and 4‐point kneeling with single arm raising (Liaghat et al. 2022b). The introduction of different phases was used as a means of progression, although no progression was used in load specifically.

### 2.6 Self‐Reported Clinical Characteristics

#### 2.6.1 Demographics

Participants were asked about age, sex, hand dominance, previous shoulder dislocation and the experience of mechanical shoulder symptoms (Liaghat et al. 2022a).

#### 2.6.2 Shoulder Function

The Western Ontario Shoulder Instability Index (WOSI) was used to assess shoulder function. WOSI was developed for patients with shoulder instability, with 21 questions covering four domains. Each question was scored on a visual analogue scale (VAS) ranging from 0 to 100, giving a total score of 0–2100, with the best possible score being 0, equivalent to no limitations. The four domains are ‘Physical symptoms’ (10 questions), ‘Sport, recreation and work’ (four questions), ‘Lifestyle’ (four questions) and ‘Emotions’ (three questions) (H. Eshoj et al. 2017). A reduction of 252 in the WOSI score was set as the Minimal Clinically Important Difference (MCID). (Kirkley et al. 2005; Linde et al. 2017).

#### 2.6.3 Shoulder Pain

The Numerical Pain Rating Scale (NPRS) was used to assess pain. The highest, lowest, and average pain levels for the past 7 days were measured with numbers from 0 to 10 (‘no pain’ to ‘worst pain imaginable’) (Breivik et al. 2008). The average pain level was used in this analysis and a reduction of 1.5 in NPRS score was set as the MCID (Vandvik et al. 2019).

#### 2.6.4 Patient Perceived Effect (Measured at 16‐Week Follow‐Up)

A Global Perceived Effect (GPE) scale was used to assess impressions of important health changes in each of the four WOSI domains (physical symptoms, sports/recreation/work, lifestyle, and emotions). The participants rated their perceived change and importance between baseline and 16‐week follow‐up assessments on a 7‐point GPE scale ranging from ‘1 ‐ worse, an important worsening’ to ‘7 ‐ better, an important improvement’ (Ingelsrud et al. 2018; Kamper et al. 2010). The domain of the physical symptoms GPE (GPEP) was included and dichotomised so that *important improvement* was equal to points 6 and 7, and *not important/worse* was equal to points 1 to 5.

### 2.7 Objectively Measured Characteristics

#### 2.7.1 Isometric Shoulder Strength

Isometric shoulder strength (Newton) was measured on the symptomatic shoulder as the average of three Maximum Isometric Voluntary Contractions (MIVC) in external rotation, internal rotation and scaption using a handheld dynamometer (IsoForce Dynamometer EVO2; Medical Device Solution AG). The average of each strength measurement was then multiplied by the joint lever and divided by bodyweight to express relative strength torque in Nm/kg. Strength change scores were calculated by subtracting the baseline Nm/kg measurements from the follow‐up Nm/kg measurements. The Nm/kg change scores were then converted to percentage change scores before being applied to the regression models.

#### 2.7.2 Demographics

Height and weight were measured during physical screening.

### 2.8 Statistics

Statistical analysis was performed as a per‐protocol, post hoc explorative analysis based on a pre‐established Statistical Analysis Plan (Christensen et al. 2022). Normative continuous data are presented as mean with Standard Deviation (SD) and number (%) unless stated otherwise. Results are presented as the unstandardised regression coefficient (b) in linear associations, and as the Odds Ratio (OR) in logistic associations, both with 95% Confidence Intervals (CI).

Analyses were performed using both linear and logistic regression models. Regression models were first performed crudely and then adjusted for covariates. Covariates were based on data availability and the evidence on how those variables may influence outcomes in patients with HSD (Friedman et al. 2018; Gartsman et al. 1998; Robinson et al. 2006), and were: age, sex, Body Mass Index (BMI), hand dominance, previous shoulder dislocation, mechanical shoulder symptoms, and assigned intervention group. This approach represented a minor deviation from the original statistical analysis plan, which specified that each covariate would first be examined in a univariate analysis, and only adjusting for those found significant in the final model. In practice, this deviation did not alter the overall findings. The level of significance was set at *p* < 0.05 for all analysis.

Model assumptions were met when examining linear regression models for linearity of variables, normality, and heteroscedasticity of residuals through inspection of Q‐Q plots, rfvplots and histograms. Likewise, logistic models were examined by a goodness of fit test showing no violations. As data analysis was exploratory using data from a previously published RCT, no sample size calculation or correction for multiple comparisons were applied. The lack of correction increases the risk of Type I error and should therefore be considered when interpreting the findings. Lastly, we performed an analysis of correlation between covariates to evaluate the risk of multicollinearity. All statistical analyses were performed with Stata (StataCorp. 2021. Stata Statistical Software: Release 17.0. College Station, TX: StataCorp LLC).

#### 2.8.1 Primary Analysis

The association between changes in objectively measured shoulder strength in (1) external rotation, (2) internal rotation and (3) scaption, and the change scores in self‐reported outcome variables (a) shoulder function and (b) shoulder pain were analysed in linear regression models. Shoulder strength was the independent variable while shoulder function and pain were the dependent variables.

The association between changes in objectively measured shoulder strength in (1) external rotation, (2) internal rotation and (3) scaption, and the dichotomised self‐reported outcome GPEP, *important improvement* or *not important/worse,* were analysed in logistic regression models. Shoulder strength was the independent variable, whereas GPEP was the dependent variable.

#### 2.8.2 Secondary Analysis

The association between changes in objectively measured shoulder strength in (1) external rotation, (2) internal rotation and (3) scaption, and the dichotomised outcome variables of MCID in (a) shoulder function and (b) shoulder pain were analysed in logistic regression models. Shoulder strength was the independent variable, while MCID in shoulder function and pain were the dependent variables. A sensitivity analysis was performed to compare the interaction between groups in the observed associations.

## 3 Results

**FIGURE 1:**
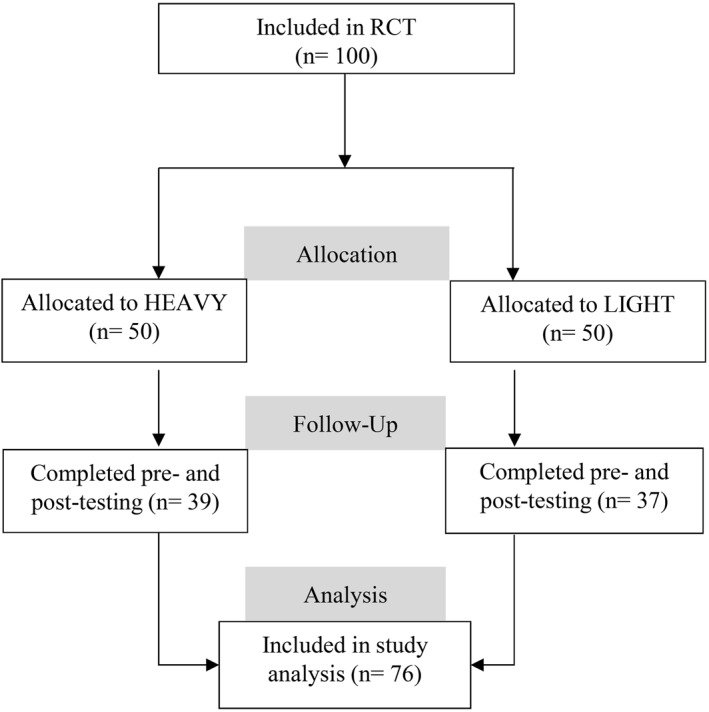
Flowchart showing the flow of participants from ‘assessed for eligibility’ to ‘included in the study analysis’, *n* = number.

Of the 100 participants recruited in the RCT, 76 completed testing at both baseline and the 16‐week follow‐up (Figure 1). Missing data was random, considering participants' age, sex, and baseline shoulder function level using WOSI.

These consisted of 58 women (76%) with a median age of 35.5 years Table 1 shows the baseline characteristics of the participants.

**TABLE 1:** Baseline demographics and characteristics of participants with Hypermobility Spectrum Disorder (HSD) and shoulder symptoms.

	LIGHT	HEAVY	All participants
	*n* = 37 (48.7%)	*n* = 39 (51.3%)	(*n* = 76)
Sex, female *n* (%)	28 (75.6)	30 (76.9)	58 (76.3)
Age, mean (SD)	37 (12)	38 (14)	37 (13)
BMI, mean (SD)	26.8 (5.0)	26.6 (5.5)	26.7 (5.2)
Right hand dominance, *n* (%)	36 (97.3)	34 (87.2)	70 (92.1)
Mechanical shoulder symptoms, *n* (%)	28 (75.7)	24 (61.5)	52 (68.4)
Previously dislocated shoulder, *n* (%)	4 (10.8)	9 (23.1)	13 (17.1)
WOSI, mean (SD)	1048 (383)	1068 (344)	1058 (361)
NPRS, mean (SD)	3.7 (2.1)	3.8 (2.2)	3.8 (2.2)

Abbreviations: % = percentages, BMI = body mass index, *n* = number, NPRS = numerical pain rating scale, SD = standard deviation, WOSI = western ontario shoulder instability index.

The HEAVY group increased strength between 10.8% and 22.0% across the three movements, while the LIGHT group increased strength between 9.2% and 14.2% (Supporting Information S1: Tables S1 and S2). Both groups reported improvements in shoulder function and reductions in pain, although these were greater in the HEAVY group (45% vs. 30% and 62% vs. 52%, respectively) (Tables S1 and S2).

### 3.1 Primary Outcomes

#### 3.1.1 Shoulder Function and Pain

**FIGURE 2:**
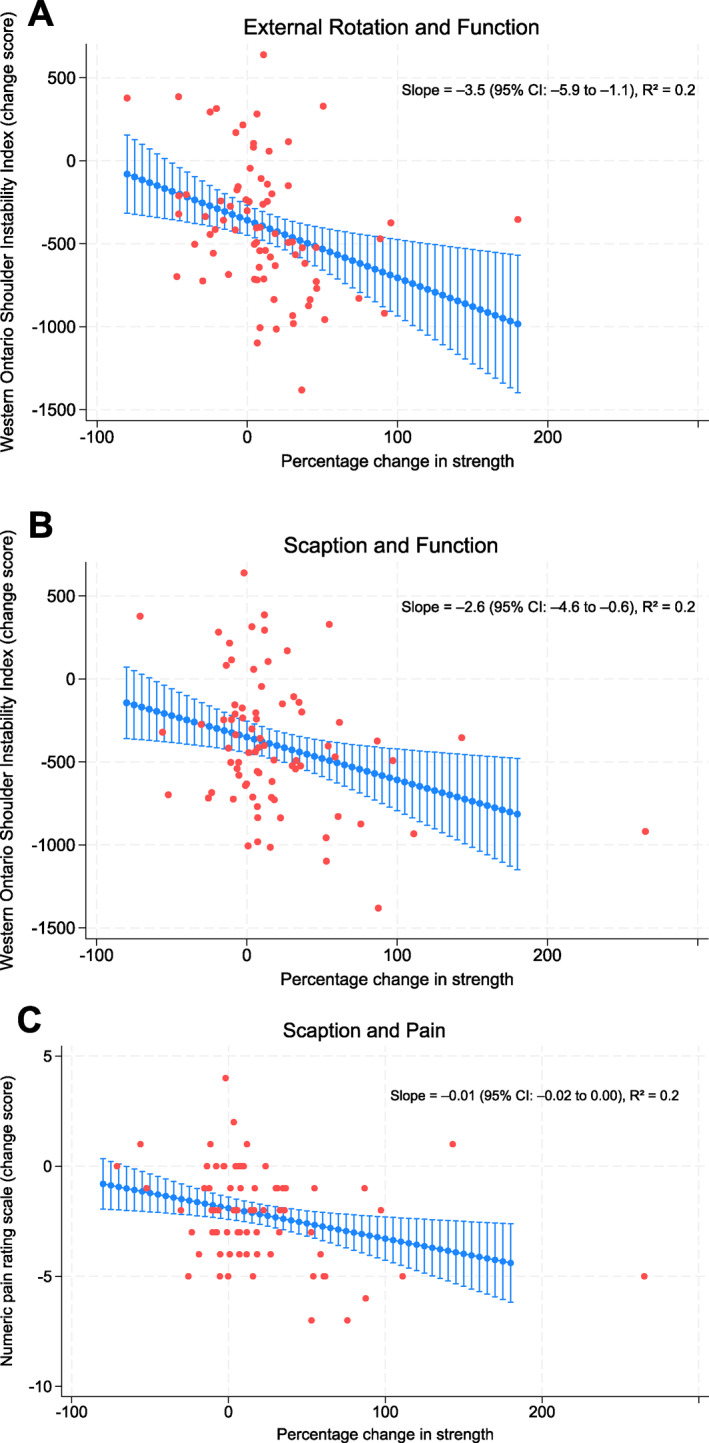
Regression lines with 95% confidence interval adjusted for age, sex, body mass index, hand dominance, previous shoulder dislocation, mechanical shoulder symptoms, and assigned intervention group. (A) Association between change in strength in external rotation and change in function measured by Western Ontario Shoulder Instability (WOSI) (lower score = better function). (B) Association between change in strength in scaption and change in function (lower score = better function). (C) Association between change in strength in scaption and change in pain measured by the Numerical Pain Rating Scale (NPRS) (lower score = less pain).

Increasing strength in external rotation and scaption were associated with improved shoulder function (WOSI adjusted slope −3.5 (95% CI −5.9; −1.1) and WOSI adjusted slope −2.6 (95% CI −4.6;−0.6), respectively) (Figure 2A,B) while scaption strength was associated with a reduction in shoulder pain (NPRS −0.01 (95% CI −0.02; 0.00)) (Figure 2C) (Table S3).

#### 3.1.2 Perceived Effect

External rotation and scaption were associated with higher odds of reporting an *important improvement,* compared to *non‐important improvement/worse,* for every 10% increase in strength (adjusted OR 1.18 (95% CI 1.00; 1.38) and 1.30 (95% CI 1.07; 1.59), respectively) (Table 2).

**TABLE 2:** Association between strength changes and patient perceived effect using logistic regression.

	Important improvement (crude)	Important improvement (adjusted^a^)
External rotation, OR (95% CI)	1.13 (0.98; 1.31)	1.18 (1.00; 1.38)*
Internal rotation, OR (95% CI)	1.10 (0.94; 1.29)	1.11 (0.93; 1.33)
Scaption, OR (95% CI)	1.23 (1.04; 1.45)*	1.30 (1.07; 1.59)*

*Note:* Values depicted as odds per 10% of increase in shoulder strength.

Abbreviations: 95% CI = 95% confidence interval, OR = odds ratio.

^a^ Adjusted for age, sex, Body Mass Index, hand dominance, previous shoulder dislocation, mechanical shoulder symptoms, and assigned intervention group.

^*^ *p*‐value significant (< 0.05).

### 3.2 Secondary Outcomes

For every 10% increase, strength in external rotation and scaption were associated with greater odds of reaching MCID in shoulder function (OR adjusted 1.34 (95% CI 1.07; 1.66) and 1.27 (95% CI 1.04; 1.55), respectively) (Table 3). No statistically significant associations were found between increasing strength in any testing position and improving the odds of reaching MCID in pain (Table 3). A post hoc subgroup analysis showed no interaction between groups in the observed associations (data not shown).

**TABLE 3:** Association between strength changes and Minimal Clinically Important Difference (MCID) in function and pain using logistic regression.

	Function (crude)	Function (adjusted^a^)	Pain (crude)	Pain (adjusted^a^)
External rotation, OR (95% CI)	1.25 (1.04; 1.49)*	1.34 (1.07; 1.66)*	1.05 (0.93; 1.20)	1.02 (0.89; 1.16)
Internal rotation, OR (95% CI)	1.02 (0.87; 1.19)	1.07 (0.89; 1.27)	1.09 (0.93; 1.28)	1.10 (0.93; 1.31)
Scaption, OR (95% CI)	1.17 (1.00; 1.37)	1.27 (1.04; 1.55)*	1.09 (0.96; 1.24)	1.08 (0.95; 1.23)

*Note:* Values depicted as per 10% of increase in shoulder strength change score.

Abbreviations: 95% CI = 95% confidence interval, Function = western ontario shoulder instability index, OR = odds ratio, Pain = numerical pain rating scale.

^a^ Adjusted for age, sex, Body Mass Index, hand dominance, previous shoulder dislocation, mechanical shoulder symptoms, and assigned intervention group.

^*^ *p*‐value significant (< 0.05).

## 4 Discussion

We found that increasing shoulder strength in external rotation and scaption may be associated with an improvement in shoulder function, while scaption may be associated with a reduction in shoulder pain. External rotation and scaption were both associated with greater odds of reporting an important improvement and greater odds of reaching MCID in shoulder function. There were no associations between any testing position (external rotation, internal rotation, scaption) and the odds of reaching MCID in pain. Neither was there any association between increasing internal rotation strength and any of the outcomes.

Patients with shoulder complaints may experience symptom relief based on a variety of exercise interventions across different shoulder pathologies (Christensen et al. 2016; Dominguez‐Romero et al. 2021; Hanratty et al. 2012; Naunton et al. 2020), yet the relationship between shoulder strength, function and pain remain unclear (Clausen et al. 2017; Erol et al. 2008; MacDermid et al. 2004; Oh et al. 2019). While our analysis appears to be the first to examine the association between changes in strength and changes in outcomes in patients with HSD and shoulder complains, Clausen et al. 2017 found no correlation between strength at baseline and outcomes in patients with subacromial impingement syndrome (SIS). In contrast, other authors have found moderate—strong correlations between baseline strength and outcomes in SIS patients (Erol et al. 2008; MacDermid et al. 2004), as well as in patients with rotator cuff tears (Oh et al. 2019).Thus, evidence on the strength‐symptom relationship seems conflicting and the association between improvements in strength and changes in symptoms has not been thoroughly examined.

Exploring the adjusted linear model in our analysis was, however, less promising, as participants needed an increase of approximately 80% in external rotation strength to reach the MCID in shoulder function. Similarly, an increase above 100% in scaption strength was needed to reach the MCID in pain. Considering that very few of the participants reached both thresholds at the 16‐week follow‐up, relying solely on strength improvements to drive a meaningful change does not seem reasonable based on our sample. However, our logistic regression models showed that improving shoulder strength increased the odds of reaching the MCID in shoulder function, as well as the odds of participants reporting a perceived *important improvement* in their physical symptoms. Although these odds were modest, they add to the story that while strength in isolation is likely not the single deciding factor, it may still be a valuable factor in facilitating a clinical improvement. Future analyses on larger sample sizes could help clarify to what extent this is the case.

When prescribing exercise interventions, health care professionals must consider both physiological and psychosocial aspects (Powell et al. 2022), as factors such as pain experience, fear‐avoidance, health‐related quality of life, and catastrophising can influence the prognosis (Engebretsen et al. 2010; Major et al. 2022; Rønnow et al. 2021). While the generalised benefit of exercise may positively influence self‐reported outcomes (Buryk‐Iggers et al. 2022; Palmer et al. 2014; Shire et al. 2017; Warby et al. 2014), dealing with the psychosocial nature of shoulder pain and function often requires a multifaceted approach that is not singularly addressed by increasing strength through resistance training. In addition, psychosocial factors may mediate the effectiveness of the exercise therapy itself (Clausen et al. 2023; Dolsø et al. 2023), highlighting the relevance of considering psychosocial barriers when planning an intervention in a clinical setting. Screening for ‘yellow flags’ as indicators may provide valuable information in guiding the rehabilitation as they are associated with long term disability (Chester et al. 2018; Stearns et al. 2021).

While our study examined the association between increases in strength and shoulder symptoms following two exercise interventions with different loading schemes, it is important to consider that progression can be more than strictly increasing load. Parameters such as volume, specific goals, training variability and recovery may all play a role in reaching the desired outcomes and improving patients' quality of life.

### 4.1 Study Limitations

A limitation of this study is that the associative nature of our analysis may introduce confounding factors and thus prevent us from making recommendations for clinical practice, as it does not provide evidence on the causal relationships between variables.

The psychosocial factors that may influence the prognosis in patients with shoulder symptoms were not included in our covariates. Lastly, correlations (*R* ^2^ values) were low in the adjusted regression models, < 0.3, showing weak strength of associations and indicating that changes in strength explain only a small portion of variance in function or pain. The estimates from these regressions may have also been influenced by outliers.

This study had strengths. Participants represented the general population of patients with HSD, matching national and international cohorts in primary care (Demmler et al. 2019; Kulas Søborg et al. 2017). Self‐reported measurements such as WOSI and NPRS are standardised tools with high validity and reliability within the population (Eshoj et al. 2017). Lastly, following the SAP made prior to this analysis allows for greater transparency of the methodology and results (Christensen et al. 2022).

## 5 Implications of Physiotherapy Practice

Increased shoulder strength may be associated with improved shoulder outcomes in patients with hypermobility and persistent shoulder symptoms although findings suggested modest clinical importance. Strength gains in scaption and external rotation appeared most beneficial for functional improvements. Further research is needed to understand the relationship between strength and symptom relief.

## Author Contributions

All authors contributed to the study concept and design. Thomas Christensen, Behnam Liaghat, and Carsten Bogh Juhl conducted the analysis. Thomas Christensen and Behnam Liaghat drafted the manuscript, and all authors participated in draughting and revising the manuscript for intellectual content and have approved the final version. Behnam Liaghat is the guarantor of the study.

## Funding

The RCT was supported by the Region of Southern Denmark, Esbjerg municipality, The Danish Rheumatism Association, Fund for Research, Quality and Education in Physiotherapy Practice, and the University of Southern Denmark. STS is currently funded by a program grant from Region Zealand in Denmark (Exercise First) and two grants from the European Union's Horizon 2020 research and innovation program, one from the European Research Council (MOBILIZE) grant agreement and the other under grant agreement (ESCAPE).

## Ethics Statement

The study was approved by the Regional Committees on Health Research Ethics for Southern Denmark (31 May 2017, S‐20170066).

## Consent

Informed consent was obtained from all participants.

## Conflicts of Interest

Co‐author Søren T. Skou reports personal fees from the Journal of Orthopaedic & Sports Physical Therapy, grants from The Lundbeck Foundation, and personal fees from Munksgaard, outside the submitted work, and being a co‐founder of Good Life with osteo‐Arthritis in Denmark (GLA:D). GLA:D is a non‐profit initiative hosted at the University of Southern Denmark aimed at implementing clinical guidelines for osteoarthritis in clinical practice. Jens Søndergaard reports receiving grants from Astra‐Zeneca, outside the submitted work.

## Permission to Reproduce Material From Other Sources

The authors have nothing to report.

## Supporting information


Supporting Information S1

## Data Availability

Data are available from the corresponding author, Behnam Liaghat, upon reasonable request.
