# Supplementary material for: Association Between Changes in Shoulder Strength and Self‐Reported Shoulder Symptoms in Patients With Hypermobility Following 16‐Weeks of High‐Load or Low‐Load Exercise: A Secondary Analysis of an RCT
Source: Physiother Res Int. 2026 Mar 18;31(2):e70197. doi: 10.1002/pri.70197 (PMC12997522; doi:10.1002/pri.70197)
Supplement: Supplementary file 1 — Supporting Information S1 [file PRI-31-e70197-s001.docx]

**SUPPLEMENTARY MATERIAL**

**Table S1**. Within group change scores in strength, function, and pain from pre- to post-intervention.

|  | HEAVY | LIGHT |
| --- | --- | --- |
| External rotation, Nm/kg (%) | 0.03 (11) * | 0.00 (0) |
| Internal rotation, Nm/kg (%) | 0.03 (9) * | 0.03 (10) * |
| Scaption, Nm/kg (%) | 0.07 (15) * | 0.02 (5) |
|  |  |  |
| Function, WOSI (%) | -480 (-45) * | -310 (-30) * |
|  |  |  |
| Pain, NPRS (%) | -2.38 (-62) * | -1.92 (-52) * |
|  |  |  |

*Values depicted as mean change scores, Nm/kg = Newton * meter per kilogram of bodyweight, % = percentage change, WOSI = Western Ontario Shoulder Instability Index, NPRS = Numerical Pain Rating Scale, * p-value significant (<0.05).*

**Table S2**. Mean (SD) baseline, follow-up and change in strength

|  | **Baseline** | **Follow-up** | **Change** |
| --- | --- | --- | --- |
| External Rotation, Nm/kg | 0.25 (0.11) | 0.26 (0.12) | 0.01 (0.07) |
| Internal Rotation, Nm/kg | 0.34 (0.16) | 0.37 (0.18) | 0.03 (0.08) |
| Scaption, Nm/kg | 0.46 (0.22) | 0.51 (0.23) | 0.05 (0.12) |
| *Nm/kg = Newton * meter per kilogram of bodyweight* | | | |

| Table S3. Association between strength changes and self-reported function and pain. Data are presented as mean (95% CI). | | |
| --- | --- | --- |
|  | **Crude** | **Adjusted †** |
| **Function** |  |  |
| External rotation | -3.4 (-5.7; -1.0)* | -3.5 (-5.9; -1.1)* |
| Internal rotation, | -0.8 (-3.8; 2.2) | -1.3 (-4.5; 1.8) |
| Scaption | -2.7 (-4.6; -0.8)* | -2.6 (-4.6; -0.6)* |
| **Pain** |  |  |
| External rotation | -0.01 (-0.02; 0.00) | -0.01 (-0.02; 0.01) |
| Internal rotation | -0.01 (-0.03; 0.00) | -0.01 (-0.03; 0.00)^a^ |
| Scaption | -0.01 (-0.02; 0.00)* | -0.01 (-0.02; 0.00)* |

Abbreviations: *Function = Western Ontario Shoulder Instability Index, Pain = Numerical Pain Rating Scale** p-value significant (<0.05).
† Adjusted for age, sex, Body Mass Index, hand dominance, previous shoulder dislocation, mechanical shoulder symptoms, and assigned intervention group
^a^Adjusted for previous shoulder dislocation
